# Angiopoietins bind thrombomodulin and inhibit its function as a thrombin cofactor

**DOI:** 10.1038/s41598-017-18912-8

**Published:** 2018-01-11

**Authors:** Christopher Daly, Xiaozhong Qian, Carla Castanaro, Elizabeth Pasnikowski, Xiabo Jiang, Benjamin R. Thomson, Susan E. Quaggin, Nicholas Papadopoulos, Yang Wei, John S. Rudge, Gavin Thurston, George D. Yancopoulos, Samuel Davis

**Affiliations:** 1 Regeneron Pharmaceuticals, Inc., 777 Old Saw Mill River Road, Tarrytown, New York, 10591 USA; 2 Feinberg Cardiovascular Research Institute, Northwestern University Feinberg School of Medicine, Chicago, Illinois USA; 3 Division of Nephrology/Hypertension, Northwestern University Feinberg School of Medicine, Chicago, Illinois USA

## Abstract

Angiopoietin-1 (Ang1) and Angiopoietin-2 (Ang2) are ligands for Tie2, an endothelial-specific receptor tyrosine kinase that is an essential regulator of angiogenesis. Here we report the identification, via expression cloning, of thrombomodulin (TM) as another receptor for Ang1 and Ang2. Thrombomodulin is an endothelial cell surface molecule that plays an essential role as a coagulation inhibitor via its function as a cofactor in the thrombin-mediated activation of protein C, an anticoagulant protein, as well as thrombin-activatable fibrinolysis inhibitor (TAFI). Ang1 and Ang2 inhibited the thrombin/TM-mediated generation of activated protein C and TAFI in cultured endothelial cells, and inhibited the binding of thrombin to TM *in vitro*. Ang2 appears to bind TM with higher affinity than Ang1 and is a more potent inhibitor of TM function. Consistent with a potential role for angiopoietins in coagulation, administration of thrombin to mice rapidly increased plasma Ang1 levels, presumably reflecting release from activated platelets (previously shown to contain high levels of Ang1). In addition, Ang1 levels were significantly elevated in plasma prepared from wound blood, suggesting that Ang1 is released from activated platelets at sites of vessel injury. Our results imply a previously undescribed role for angiopoietins in the regulation of hemostasis.

## Introduction

Tie2 (*TEK*) is an endothelial cell specific receptor tyrosine kinase that is absolutely required for embryonic development^1–3^. Tie2-deficient mice exhibit defects in cardiac development as well as vascular abnormalities, and die between embryonic days 9.5 and 12.5^1,3^. Tie2 activity is regulated by the angiopoietin family of ligands (Ang1 (*ANGPT1*)^4^, Ang2 (*ANGPT2*)^5^ and Ang4 (*ANGPT4*)^6^ in humans), of which Ang1 and Ang2 are the most well characterized. Angiopoietin/Tie2 signaling is essential for the proper formation and function of both blood and lymphatic vessels^7,8^.

Ang1 is a Tie2 agonist expressed primarily by perivascular cells, and Ang1-deficient mice have vascular defects that closely resemble those observed in Tie2-deficient mice^4,9^. Ang1/Tie2 signaling is believed to be important for blood vessel maturation and stabilization, for example by promoting endothelial cell survival and by decreasing blood vessel permeability^10–15^.

In contrast to Ang1, Ang2 was initially identified as an antagonist of Tie2 that is expressed by the endothelial cells in remodeling blood vessels^5^. However, several subsequent studies showed that Ang2 is capable of activating Tie2^16–18^, and the mechanism of Ang2 signaling remains controversial^19–24^. Ang2 is highly expressed in the vasculature of many tumor types and it appears to play an important role in tumor angiogenesis, possibly as a Tie2 agonist^7,8,25^. In addition to tumors, Ang2 expression is increased in a number of inflammatory settings, where it is proposed to have pro-inflammatory effects and/or to promote vascular destabilization and leak by inhibiting Tie2 function^26–38^.

While overwhelming evidence indicates that angiopoietins regulate Tie2 activity, several reports suggest that angiopoietins can also modulate cell function via interactions with other proteins, for example integrins^32,39–46^. While these reports are intriguing, in many cases direct angiopoietin/integrin binding has not been convincingly demonstrated and the functional significance of these interactions remains unclear. These considerations motivated us to search for additional angiopoietin binding partners, using an expression cloning strategy.

In this report we describe the unexpected identification of TM as a receptor for angiopoietins. Thrombomodulin is an endothelial cell surface protein and an essential negative regulator of coagulation through its function as a cofactor in the thrombin-mediated generation of activated protein C (APC)^47–51^. We show that both Ang1 and Ang2 inhibit TM function in cultured endothelial cells, possibly by interfering with thrombin/TM binding. These findings suggest the possibility that angiopoietins have a previously unappreciated role in the regulation of hemostasis.

## Results

### Thrombomodulin is a receptor for angiopoietins

cDNA expression libraries of human umbilical vein endothelial cells (HUVECs) and the endothelial-like cell line EA.hy926 were transfected into dishes of COS cells and screened with the chimeric molecule Ang1-FD-Fc, consisting of the Tie2-binding fibrinogen-like domain (FD) of Ang1 fused to a human Fc tag^52^. Stained cells were rescued, and plasmid DNA extracted from the stained cells was progressively enriched until isolated clones were obtained. Surprisingly, out of eight independent clones, only one of the associated cDNAs coded for Tie2. The other seven clones, differing only in the length of untranslated regions, contained cDNAs encoding TM, a cell surface protein highly expressed in endothelial cells^51^. Only rare cells were stained on dishes of library-transfected COS cells, indicating that Ang1-FD-Fc binding to Tie2 and TM is highly specific. Further staining experiments revealed that like Ang1-FD-Fc, Ang2-FD-Fc could also bind to COS cells expressing either Tie2 or TM. In contrast, Ang4-FD-Fc could bind to COS cells expressing TIE2, but not to those expressing TM (data not shown). This provides evidence that the interactions between angiopoietins and TM are specific to Ang1 and Ang2.

**Figure 1:**
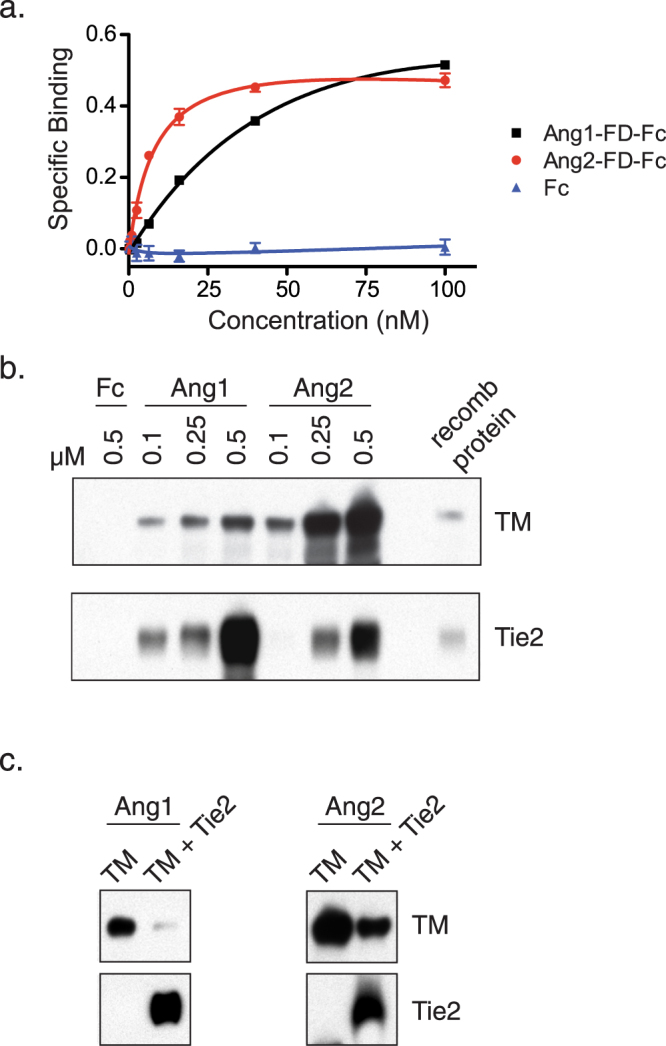
Ang1 and Ang2 interact with TM via their fibrinogen-like domains. (**a**) Ang1 and Ang2 bind saturably to COS cells overexpressing TM. Cells were incubated with varying concentrations of Ang1-FD-Fc or Ang2-FD-Fc, and binding was assessed with an alkaline phosphatase-conjugated secondary antibody. Specific binding, given in OD405 units (see Methods), is defined as the difference between binding to TM-transfected COS cells and binding to COS cells transfected with empty vector. (**b**) The soluble extracellular domains of either TM or Tie2 (100 nM) were incubated on ice for 60 minutes with BowAng1 (Ang1), BowAng2 (Ang2) or human Fc control protein at the indicated concentrations (BowAng1 and BowAng2 are chimeric angiopoietins that contain human Fc, see Methods for details). The Fc-containing proteins were then collected on protein A/G beads and co-precipitation of TM or Tie2 was assessed by western blot. (**c**) BowAng1 (Ang1) or BowAng2 (Ang2) (100 nM) were incubated with the soluble extracellular domain of TM (100 nM) in the absence or presence of the soluble extracellular domain of Tie2 (2 μM). The Fc-containing BowAng1 and BowAng2 were then collected on protein A/G beads and co-precipitation of TM and Tie2 was assessed by western blot.

Both Ang1-FD-Fc and Ang2-FD-Fc, but not a control Fc protein, bound saturably to COS cells engineered to express TM (Fig. 1a), with apparent binding affinities of ~88 nM for Ang1-FD-Fc and ~8.3 nM for Ang2-FD-Fc. To determine whether angiopoietins bind directly to TM, we tested the ability of the chimeric angiopoietins BowAng1 and BowAng2 (see Methods for details) to associate with the purified soluble extracellular domain of TM. As a positive control, we assessed binding of BowAng1 and BowAng2 to the soluble extracellular domain of Tie2. Both BowAng1 and BowAng2, but not control Fc protein, associated with TM, with estimated EC_50_ values in the hundreds of nM (Fig. 1b). Consistent with the cell surface binding assay, BowAng2 bound to TM significantly more tightly than did BowAng1 (Fig. 1b). Since soluble TM is monomeric, this assay provides a measure of monomer-monomer interactions between TM and one fibrinogen-like domain of BowAng1 or BowAng2. Thus, it is free of avidity effects inherent in the binding of dimeric forms of angiopoietins to cell surface TM (Fig. 1a), so it is not unexpected that lower apparent affinities are observed. The relatively low apparent affinity of angiopoietins for monomeric Tie2 observed here (estimated EC_50_ values in the hundreds of nM) is consistent with the previously reported avidity effects that are characteristic of angiopoietin-Tie2 binding^52^. Although the multimeric nature of angiopoietins raises the possibility that they induce the formation of complexes on the surface of cells that include both Tie2 and TM, co-precipitation experiments failed to provide evidence for the existence of such complexes (data not shown).

To determine whether the TM and Tie2 binding sites on the angiopoietins overlap, we tested the ability of soluble Tie2 extracellular domain to compete with TM for binding to angiopoietins. BowAng1 or BowAng2 (100 nM) were incubated with TM (100 nM) in the absence or presence of an excess of Tie2 (2.0 μM). As shown in Fig. 1c, Tie2 competed with TM for binding to BowAng1 and BowAng2, suggesting that the binding sites on the angiopoietins for TM and Tie2 do overlap. However, Tie2 was less effective at competing with TM for binding to BowAng2, consistent with the observation that BowAng2 appears to bind TM more tightly than does BowAng1 (Fig. 1b). Furthermore, in staining experiments with COS cells expressing TM, we failed to observe binding of an Ang2-FD-Fc triple mutant that does not bind to Tie2^53^, in which the mutated residues (F468A/Y474A/Y475A) are known to be located at the Ang2/Tie2 interface^54^ (data not shown). This observation provides further support for the notion that residues at the angiopoietin/Tie2 binding interface are also required for binding to TM.

### Angiopoietins inhibit thrombomodulin function

The finding that angiopoietins bind to TM led us to ask whether the angiopoietins could modulate the thrombin/TM dependent activation of protein C and/or TAFI (we think it unlikely that angiopoietin binding to TM directly regulates intracellular signaling, given the absence of known signaling motifs in the short cytoplasmic domain of TM). Addition of protein C or TAFI to cultured endothelial cells in the presence of thrombin results in cleavage of these substrates in a TM-dependent fashion^55–57^. Several previous reports have used cultured endothelial cells to assess the effects of various proteins (e.g., the platelet α-granule protein platelet factor 4 (PF4)) on the activity of the thrombin/TM complex^57^.

**Figure 2:**
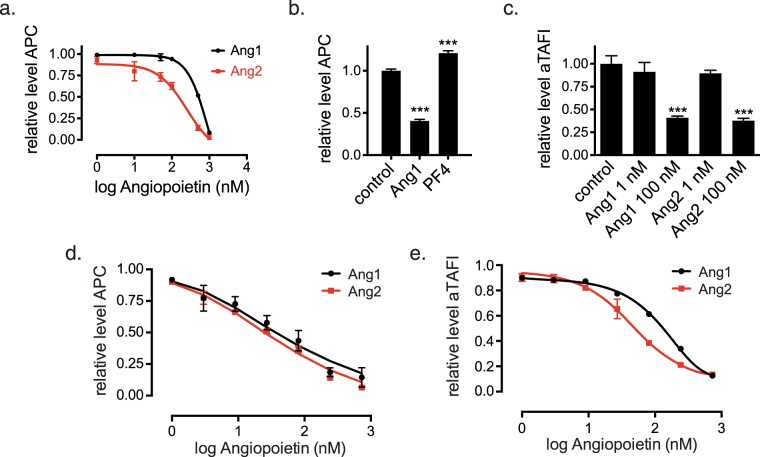
Ang1 and Ang2 inhibit TM-dependent formation of APC and TAFIa. (**a**) HUVECs were incubated with protein C and thrombin plus either 1 μM of Fc control protein or the indicated concentrations of native Ang1 or Ang2 for 60 minutes. Cell supernatants were then assayed for APC levels. The graph depicts the relative amounts of APC formation (compared to APC formation in the presence of the Fc control protein) as a function of angiopoietin concentration. The error bars represent the SD, n = 3. (**b**) HUVECs were incubated with protein C and thrombin plus either Fc control protein (0.5 μM), native Ang1 (0.5 μM) or PF4 (10 μg/ml, ~1.3 μM) for 60 minutes and cell supernatants were then assayed for APC levels. The graph depicts the relative amounts of APC generated (the amount of APC generated in the presence of Fc control protein was assigned a value of 1.0). The error bars represent the SD, n = 3. Relative levels of APC in the Ang1 and the PF4 treatment groups were significantly different from the control group (***P < 0.001, one-way ANOVA with Tukey’s multiple comparisons test). (**c**) HUVECs were incubated with protein C and thrombin plus either 1  μM of Fc control protein or the indicated concentrations of native Ang1 or Ang2 for 60 minutes. Cell supernatants were then assayed for TAFIa levels. The graph depicts the relative amounts of TAFIa generated (the amount of TAFIa generated in the presence of Fc control protein was assigned a value of 1.0). The error bars represent the SD, n = 3. Relative levels of TAFIa in the Ang1 100 nM and the Ang2 100 nM treatment groups were significantly different from the control group (***P < 0.001, one-way ANOVA with Tukey’s multiple comparisons test). (**d**,**e**) COS cells overexpressing TM were incubated with the indicated concentrations of native Ang1 or Ang2 for 30 minutes, and then assayed for APC (**d**) or TAFIa (**e**) formation upon addition of thrombin plus protein C or TAFI. The graphs depict relative APC or TAFIa formation as a function of angiopoietin concentration (the amounts formed in the absence of angiopoietin were assigned a value of 1.0).

We first tested the effects of native Ang1 and Ang2 on APC formation in cultured HUVECs. Both Ang1 and Ang2 substantially inhibited APC formation (relative to the amount generated in the presence of 1 μM of a control protein), with IC_50_ values in the hundreds of nM (Fig. 2a). Consistent with its higher affinity for TM, Ang2 was a more potent inhibitor of APC production than was Ang1 (Fig. 2a). As an additional control for the specificity of the assay, we showed that in contrast to angiopoietins, addition of PF4 to the HUVECs modestly increased APC formation (Fig. 2b), as demonstrated previously^57^. Similar to their effect on formation of APC, native Ang1 and Ang2 at 100 nM significantly inhibited TAFIa formation in HUVECs (Fig. 2c). These experiments indicate that in addition to binding TM, native Ang1 and Ang2 inhibit TM function in endothelial cells.

In COS cells overexpressing TM, but lacking Tie2 expression, angiopoietins significantly inhibited both APC and TAFIa formation, indicating that the inhibition does not require Tie2 (Fig. 2d and e). In contrast to their effect in HUVECs, angiopoietin-mediated inhibition in COS cells was observed even at low nM concentrations, presumably reflecting enhanced, avidity-driven angiopoietin binding to overexpressed TM. It also seems possible that in HUVECs, the endothelial Protein C receptor (EPCR) makes blockade of APC formation more difficult than in COS cells. EPCR is expressed at high levels in HUVECs and binds Protein C with high affinity^58^, promoting delivery of protein C to the thrombin/TM complex, thus amplifying its activity^59^. In this context, it is interesting to note that, in contrast to HUVECs, EPCR is expressed at low levels in microvasculature^60^.

**Figure 3:**
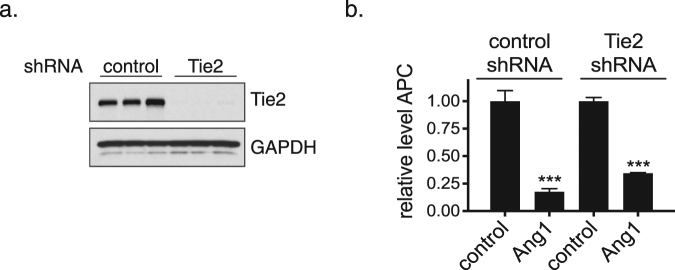
Inhibition of APC formation by Ang1 is independent of Tie2. HUVECs were infected with adenoviruses encoding either a control shRNA or a Tie2 specific shRNA. At 3 days after infection, cells were either lysed for western blot to demonstrate Tie2 knockdown (**a**) or used to test the effect of native Ang1 on APC formation in the presence of thrombin plus protein C (**b**). The graph depicts the relative amounts of APC generated in the presence of 1 μM Fc control protein or native Ang1 (the amount of APC generated in the presence of Fc control protein was assigned a value of 1.0). The error bars represent the SD, n = 3. Ang1 significantly inhibited APC formation in cells treated with control shRNA or Tie2 shRNA (***P < 0.001, one-way ANOVA with Tukey’s multiple comparisons test).

To further confirm that the ability of angiopoietins to inhibit the function of the thrombin/TM complex in endothelial cells is independent of Tie2, we knocked down Tie2 expression in HUVECs with a specific shRNA. Despite almost complete inhibition of Tie2 expression by the shRNA (Fig. 3a), Ang1 was still capable of inhibiting APC formation (Fig. 3b), indicating that Tie2 is not required for this effect.

**Figure 4:**
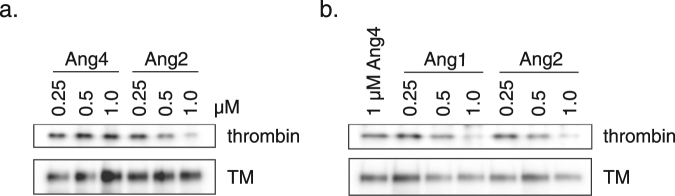
Ang1 and Ang2 inhibit binding of thrombin to TM. **(a**,**b)** Soluble extracellular domain of TM at 100 nM was incubated with 5 nM thrombin in the presence of the indicated concentrations of native Ang1, native Ang2 or native Ang4. Following collection of TM on Ni-NTA beads, the beads were washed and bound proteins were eluted in SDS sample buffer. Western blots were performed to assess pull-down of TM and co-precipitation of thrombin.

The observation that angiopoietins inhibit formation of APC and TAFIa led us to ask whether Ang1/Ang2 could directly interfere with the thrombin/TM interaction. Since Ang4 does not bind TM, we used it as a negative control in these experiments. As shown in Fig. 4a and b, both Ang1 and Ang2 significantly inhibited (relative to Ang4 control) the association of thrombin with soluble TM. For example, Ang2 at 1 μM inhibited formation of the thrombin/TM complex by ~80% (Fig. 4a). The requirement for high concentrations (>0.5 μM) of Ang1/Ang2 to inhibit thrombin/TM binding is consistent with the finding that high concentrations of Ang1/Ang2 (hundreds of nM) are necessary to inhibit the function of the thrombin/TM complex in cultured endothelial cells (Fig. 2). These findings suggest that Ang1/Ang2 can inhibit formation of a stable complex of thrombin with TM, and that this is one mechanism by which angiopoietins interfere with generation of APC and TAFIa. However, given that both Protein C and TAFI are known to interact with thrombomodulin^61,62^, it is possible that angiopoietins disrupt these interactions as well. The determinants of overall response may be a combination of factors, including other components of the system (for example, as mentioned above, EPCR).

### Ang1 is released from activated platelets *in vivo*

**Figure 5:**
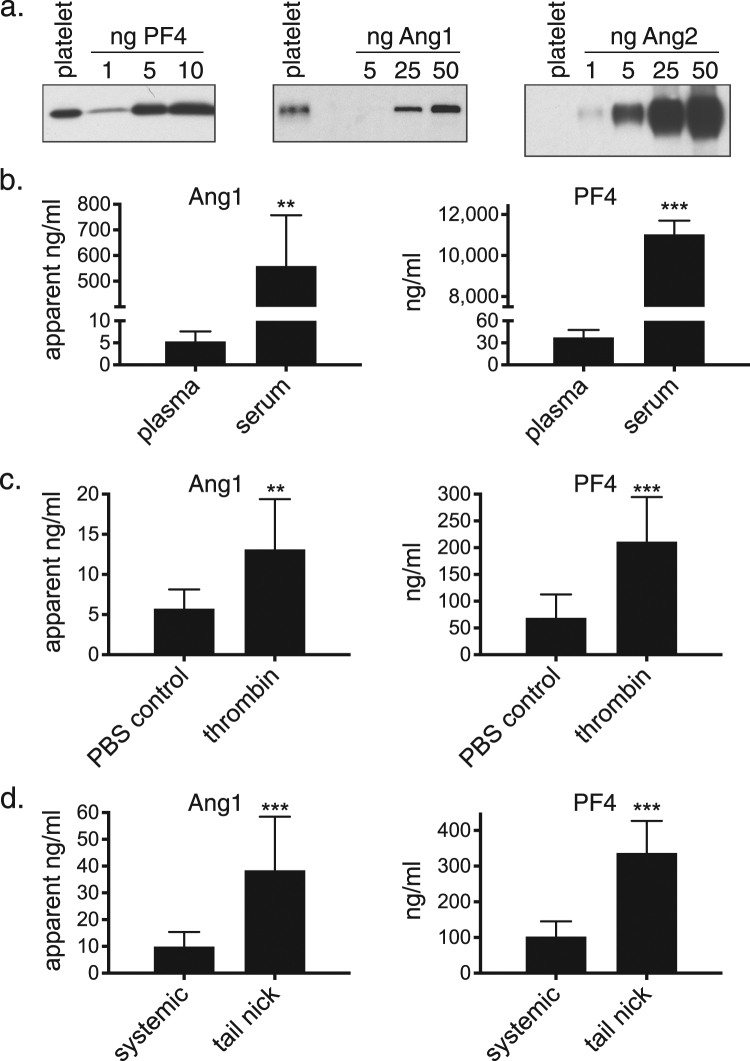
Ang 1 is present in platelets and is released upon activation. (**a**) Platelets were isolated from C57BL/6 mice as described in the Methods and lysed in SDS sample buffer. Aliquots of platelet lysate representing ~1.6 × 10^6^ platelets were run on SDS gels along with the indicated amounts (ng) of mouse PF4, human Ang1 or human Ang2 to allow estimation of the levels of these proteins in the platelet lysate. The Ang1 and Ang2 antibodies recognize both the human and mouse proteins. (**b**) Blood was collected from C57BL/6 mice by cardiac puncture and used to prepare either plasma or serum. Ang1 and PF4 levels were then determined by ELISA as described in the Methods. Ang1 levels are given as apparent concentrations since the standard curve used to estimate Ang1 concentration was generated with human Ang1 protein. Bars represent the mean and SD, n = 4. Both Ang1 and PF4 levels were significantly higher in serum (**P < 0.01; ***P < 0.001, *t*-test). (**c**) C57BL/6 mice were injected via the tail vein with PBS or 10 units of thrombin. After 10 minutes, blood was collected by cardiac puncture and plasma was prepared. Plasma levels of Ang1 and PF4 were determined by ELISA. Bars represent the mean and SD (n = 9 for PBS, n = 8 for thrombin). The levels of both Ang1 and PF4 were significantly higher in the thrombin-treated samples (**P < 0.01; ***P < 0.001, *t*-test). (**d**) Blood was collected from C57BL/6 mice either by cardiac puncture (systemic) or by tail nick as described in the Methods section. Plasma was prepared and Ang1 and PF4 levels were determined by ELISA. Bars represent the mean and SD (n = 9 for systemic samples, n = 10 for tail nick samples). The levels of both Ang1 and PF4 were significantly higher in the tail nick samples (***P < 0.001, *t*-test).

It has been reported that human platelets contain Ang1 and release it upon stimulation with thrombin *in vitro* ^63^. To assess whether Ang1 is released from platelets *in vivo*, we first confirmed that Ang1 is present in mouse platelets. As shown in Fig. 5a, we detected Ang1 protein in mouse platelets at levels (~30 ng/10^6^ platelets) similar to those reported in human platelets (~55 ng/10^6^ platelets)^64^. As a positive control, we show the presence of PF4 in our platelet lysate at ~3 ng/10^6^ platelets, similar to the estimated level of PF4 in human platelets (~12 ng/10^6^ platelets)^65^. In contrast, we did not detect any Ang2 in mouse platelets (Fig. 5a). The polyclonal antibody we used to detect Ang2 was raised against a peptide that is identical in human/mouse Ang2 and readily detects Ang2 in mouse tissue lysates by western blot. Thus, we are confident that the inability to detect Ang2 in our mouse platelet lysate is a meaningful result. Interestingly, while Ang2 is apparently not present in platelets, it is present in endothelial cell Weibel-Palade bodies (WPB) and is released upon stimulation with thrombin *in vitro* ^66^, suggesting the possibility that Ang2 is released from activated endothelial cells at sites of vessel injury.

Consistent with Ang1 release from platelets upon degranulation *ex vivo*, Ang1 levels were ~100-fold higher in serum than in plasma (Fig. 5b). Similarly, PF4 levels were hundreds of fold higher in serum, providing a positive control (Fig. 5b). Consistent with our data showing the absence of Ang2 in platelets, Ang2 levels in plasma and serum from healthy volunteers are similar (<5 ng/ml)^67,68^. To determine whether Ang1 is released from activated platelets *in vivo*, we measured plasma levels of Ang1 (and PF4 as a positive control) 10 minutes after intravenous administration of thrombin to mice. As shown in Fig. 5c, plasma levels of both Ang1 and PF4 were significantly higher following acute thrombin treatment, consistent with release of both of these factors from activated platelets. Our results are similar to previous reports showing increased PF4 plasma levels following thrombin infusion in mice^69^.

To assess whether Ang1 is released from platelets in response to blood vessel injury, we measured Ang1 levels in plasma prepared from blood collected at the site of tail nicks in mice. Both Ang1 and PF4 levels were significantly higher in plasma prepared from tail nick blood than in plasma prepared from systemic blood (collected via cardiac puncture) (Fig. 5d), suggesting release of Ang1 from platelets at the site of injury. To ensure that the higher levels of Ang1 in the tail nick samples did not simply reflect differences in the plasma preparation procedures, the cardiac puncture blood samples were incubated in heparinized capillary tubes for 4–5 minutes before proceeding with plasma isolation to mimic the procedure for the tail nick samples (see Methods for details). Thus, Ang1 is present in platelets and appears to be released upon platelet activation *in vivo*, consistent with a potential role for Ang1 in regulation of hemostasis.

To begin to investigate whether Ang1 plays a role in coagulation, bleeding times in wild-type and conditional *Angpt1* knockout mice^70^ were compared. As shown in Supplementary Fig. 1, no significant difference in bleeding times was observed between *Angpt1* knockout mice and littermate controls. However, due to its inherent variability, this is not a particularly sensitive assay. In addition, Ang2 (which appears to be a more potent inhibitor of TM than Ang1) might compensate for the absence of Ang1. Thus, a role for the angiopoietins as regulators of TM function *in vivo* remains to be established.

## Discussion

We have shown in this report that both Ang1 and Ang2 bind to TM and significantly inhibit the generation of APC and TAFIa by the thrombin/TM complex in cultured endothelial cells. These findings, coupled with the localization of angiopoietins to cellular compartments that contain well-established regulators of hemostasis, platelet granules for Ang1^63^ and Weibel-Palade bodies for Ang2^66^, suggest the possibility that angiopoietins play a role in the regulation of coagulation.

Our functional studies indicate that fairly high concentrations of angiopoietins (e.g., at least 100 nM of Ang2) are required to significantly inhibit TM-dependent APC formation in HUVECs (Fig. 2a). Importantly, these concentrations are much higher than the concentrations of angiopoietins in plasma and the concentrations needed to maximally activate Tie2 in cultured endothelial cells (~5 nM). Thus, inhibition of TM function on the endothelial cell surface may be restricted to sites of very high local concentrations of angiopoietins, for example at sites of platelet degranulation (Ang1) or Weibel-Palade body exocytosis (Ang2). The requirement for such high levels of angiopoietins to achieve a significant effect on TM function likely precludes widespread angiopoietin-dependent inhibition of TM, which might lead to thrombosis. Similarly, the ability of PF4 to regulate thrombin/TM-dependent APC formation in cultured endothelial cells requires high levels of PF4 (0.1–0.2 μM)^57^, likely restricting this effect to sites of platelet activation, where high concentrations of PF4 are expected.

Our binding data (Fig. 1) indicate that the region of the angiopoietins necessary for binding to TM is in close proximity to the region involved in Tie2 binding, suggesting that an angiopoietin fibrinogen domain cannot simultaneously bind to both Tie2 and TM. It is not clear whether the apparent ability of Tie2 and TM to compete with each other for binding to angiopoietins has any functional significance. Tie2 in normal tissues is thought to be regulated primarily by pericyte-derived Ang1 on the abluminal surface of endothelial cells^71^, while TM acts at the luminal surface. Thus, it is possible that the respective angiopoietin receptors do not substantially interfere with one another.

Our experiments with cultured endothelial cells indicate that angiopoietins can modulate the function of TM. However, it remains unclear whether angiopoietins regulate hemostasis *in vivo*. While *Angpt1*-deficient mice die early in gestation, generation of conditional *Angpt1* knockout mice has been described^70^. Global deletion of *Angpt1* after E13.5 resulted in no overt phenotype (unless the mice were challenged by injury) and no defects in coagulation were reported^70^, although identifying such abnormalities would likely require more detailed investigation. Similarly, while no defects in coagulation in *Angpt2* knockout mice have thus far been reported^29,72^, focused experiments will be required to adequately address this issue. Furthermore, since both Ang1 and Ang2 can regulate TM function, uncovering an effect on coagulation may require analysis of mice in which both the *Angpt1* and *Angpt2* genes have been disrupted.

An agent known as trebananib (formerly AMG386) that binds to both Ang1 and Ang2 and inhibits their interaction with Tie2^73^ has been tested in multiple clinical trials in cancer and has been administered to a fairly large number of patients^74,75^. Assuming that trebananib also blocks binding of angiopoietins to TM (as would be predicted based on the observation that the TM and Tie2 binding sites on angiopoietins overlap), one might anticipate that coagulation deficits would be observed in trebananib-treated patients if the angiopoietins play an important role in the regulation of hemostasis. To our knowledge, coagulation disorders have not been reported as a significant adverse effect of trebananib treatment^74–76^. However, given that high levels of angiopoietins might be rapidly delivered to sites of vessel injury via platelet degranulation and/or Weibel-Palade body exocytosis, the binding of angiopoietins to TM might be very difficult to inhibit pharmacologically.

In addition to their roles in coagulation/fibrinolysis, both APC and TAFIa have been proposed to have anti-inflammatory functions. For example, APC has been shown to decrease the responsiveness of endothelial cells to TNFα stimulation^77^ and to play a protective role in a variety of injury models, including endotoxin challenge^78–80^. Furthermore, TAFIa can cleave and inactivate several inflammatory mediators, for example bradykinin and C5a^81,82^. While the effects of Ang2 in various inflammatory settings might be due solely to modulation of Tie2 signaling^7^, our findings raise the possibility that these effects are at least partly attributable to inhibition of TM-dependent APC and/or TAFIa formation.

Another possible connection between angiopoietin/Tie2 signaling and coagulation is suggested by the recent finding that APC binds directly to Tie2 and competes with angiopoietins for binding^83^. While the functional significance of APC binding to Tie2 remains to be determined, it appears that the regulation of both Tie2 and TM during inflammation/coagulation may be more complex than previously appreciated.

The unexpected discovery that angiopoietins interact directly with TM and modulate its function raises many interesting new questions. Future studies will be required to further explore the involvement of these interactions in the processes of coagulation, fibrinolysis, and inflammation, and in associated pathologies, and potentially to identify new avenues for therapeutic intervention.

## Methods

### HUVEC/EA.hy926 library screen

cDNA libraries were constructed in the expression vector pJFE with mRNA isolated from HUVECs and from EA.hy926 cells, using a kit (Invitrogen). Screening was performed as previously described^84^. Ang1-FD-Fc, consisting of the Tie2-binding fibrinogen-like domain (FD) of Ang1 fused to a human Fc tag^52^, was used at a concentration of 1 μg/ml.

### Binding of angiopoietins to TM expressing cells

COS cells in 48-well plates were transfected with an expression vector containing TM, or with empty vector as a control. Two days after transfection, cells were incubated with varying concentrations of Ang1-FD-Fc or Ang2-FD-Fc^52^ in 200 μl binding buffer (PBS/10% calf serum) for 30 minutes at room temperature. After two PBS washes, cells were fixed in cold methanol, followed by incubation with binding buffer containing a 1:1000 dilution of alkaline phosphatase-conjugated goat anti-human Fc (Promega) for 30 minutes. After two more PBS washes, 200 μl PNPP substrate (Sigma) was added. Reactions were stopped after 5 min by addition of 50 μl 3 N NaOH, and the OD405 was taken as a measure of binding. Measurements were in quadruplicate. There was significant variability in these measurements, likely a consequence of avidity effects resulting from variable transfection efficiencies. Some nonspecific binding of Ang1-FD-Fc to the empty vector-transfected cells was observed at high concentrations, but was less than 20% of binding to cells expressing TM.

### Interaction of angiopoietins with soluble TM

Binding of TM to angiopoietins was assessed using engineered versions of Ang1 and Ang2 known as Ang-F1-Fc-F1 (BowAng1) and Ang-F2-Fc-F2 (BowAng2)^52^. BowAng1 and BowAng2 contain the Ang1 or Ang2 fibrinogen domain followed by human Fc and another fibrinogen domain, so that following Fc-mediated dimerization, the proteins contain 4 angiopoietin fibrinogen domains^52^. The soluble extracellular domain of human TM (R&D Systems) or the soluble extracellular domain of human Tie2 (produced at Regeneron) at 100 nM were incubated with BowAng1 or BowAng2 at 100, 250 or 500 nM or with 500 nM human Fc control protein in binding buffer (0.15 M NaCl, 20 mM Tris pH 7.5, 2 mM CaCl_2_, 0.1% Triton X-100, 1 mg/ml BSA) on ice for 60 minutes. In experiments testing the ability of Tie2 to compete with TM for binding to angiopoietins, 100 nM BowAng1 or BowAng2 was incubated with 100 nM TM in the absence or presence of 2 μM Tie2 extracellular domain. Following incubation, BowAng1, BowAng2 or human Fc were pulled down by incubation with 25 μl protein A/G beads (Santa Cruz Biotechnology) for 60 minutes. The beads were then washed with binding buffer and proteins were eluted by heating in SDS sample buffer and then resolved on an SDS gel. Co-precipitation of TM and Tie2 was assessed by western blot with an anti-TM monoclonal antibody (Santa Cruz Biotechnology (D-3), used at 1:500 dilution) or a monoclonal anti-Tie2 antibody (clone 33.1^85^).

### Effect of Ang1 and Ang2 on TM-dependent APC and TAFIa formation

#### HUVEC assays

APC formation on HUVEC monolayers was assessed using a modified version of a previously described method^57^. 50,000 HUVECs (VEC Technologies) were plated in 48-well plates in 200 μl of MCDB131 complete medium. The next day the cells were washed three times with reaction buffer (HBSS + 10 mM HEPES pH 7.4 + 1 mg/ml BSA) and then incubated for 60 minutes at 37 °C with 100 μl of reaction buffer containing 0.2 μM protein C (Enzyme Research Labs), 0.1 U/ml thrombin (Sigma) and native human Ang1 (R&D Systems), native human Ang2 (R&D Systems), human PF4 (R&D Systems) or a control Fc-containing protein at the concentrations indicated in the Figure Legends. Following incubation, 90 μl of buffer from each well was transferred to a 96-well plate and incubated with the thrombin inhibitor hirudin (Sigma) at 25 U/ml for 10 minutes at 37 °C. The chromogenic APC substrate S-2366 (Chromogenix) was then added at 0.2 mM and cleavage of S-2366 was followed by monitoring the change in OD 405 nm. The OD 405 nm at ~10 minutes after S-2366 addition (the OD 405 nm was still increasing linearly at this time point) was used to assess the relative levels of TM-dependent APC formation in each sample (after subtracting the value observed when the procedure was carried out with no HUVECs in the wells).

To assess TAFIa formation, HUVECs under the same culture conditions described above were incubated at 37 °C for 60 minutes with 100 μl of reaction buffer containing 0.2 μM TAFI (Enzyme Research Labs), 1 U/ml thrombin and native human Ang1, native human Ang2 or a control Fc-containing protein at the concentrations indicated in the Figure Legend. Following incubation, cell supernatants were used to measure TAFIa activity using the fluorogenic ACTIFLUOR TAFIa Activity Assay (American Diagnostica).

#### COS cell assays

COS7 cells transfected with expression vectors for TM or control empty vector were seeded, one day after transfection, into 48-well plates (~80,000 cells/well). The next day, cells were washed three times with reaction buffer (HBSS + 10 mM HEPES + 1 mg/ml BSA) and then incubated with 100 μl of reaction buffer containing various concentrations of native human Ang1 or Ang2 for 30 minutes at 4 °C. 0.2  μM protein C and 0.1 U/ml thrombin were then added and incubated for 60 minutes at 37 °C. Following incubation, the reaction was stopped with hirudin and APC levels were determined, as above. For activated TAFI formation, COS cells transfected with expression vectors for TM or control empty vector in 10 cm dishes were rinsed in PBS, collected using a spatula, disaggregated by trituration, and pelleted. Cells were resuspended in reaction buffer (HBSS + 10 mM HEPES pH7.4 + 1 mg/ml BSA, ~80,000 cells/reaction) and incubated with 72 μl of reaction buffer containing various concentrations of native human Ang1 or Ang2 for 30 minutes at 4 °C. Following this, 0.2  μM TAFI and 1 U/ml thrombin were added in a final volume of 80 μl and incubated for 30 minutes at 22 °C. Cells were then pelleted and 75 μl of the supernatants were transferred to a 96-well plate. 25 μl of 2 mM 5,5′-dithiobis-(2-nitrobenzoic acid) (Sigma) and 50 μl of a thiol-releasing TAFI substrate (R2 reagent, Pefakit TAFI, Pentapharm) were then added, and the OD 405 nm at 10 minutes was used to determine TAFI activity.

### Knockdown of Tie2 with shRNA

HUVECs were infected with adenoviruses (~30 pfu/cell) encoding either a non-silencing control shRNA or a Tie2 specific shRNA as described^12^. The sequence of the Tie2 shRNA was: 5′-TGAAGTACCTGATATTCTA-3′ (nucleotides 946–964 in the human Tie2 (*TEK*) cDNA, NM_000459). At 2 days after infection, cells were trypsinized and seeded into 6-well plates to assess Tie2 knockdown by western blot or into 48-well plates for APC assay the next day. The APC assay was done as described above (except that thrombin was present at 1 U/ml), in the presence of 1 μM control protein or Ang1. Western blot for Tie2 was performed with the monoclonal antibody clone 33.1^85^.

### Effect of Ang1 and Ang2 on the association of thrombin with TM

Soluble TM extracellular domain (with a 6-His tag) at 100 nM was incubated with 5 nM thrombin in the presence of various concentrations (0.25, 0.5 or 1.0 μM) of native Ang1, Ang2 or Ang4 (R&D Systems) in binding buffer (0.15 M NaCl, 20 mM Tris pH 7.5, 0.1% Triton X-100, 2 mM CaCl_2_, 1 mg/ml BSA) for 60 minutes on ice. TM was then pulled down by incubation for 60 minutes with 20 μl Ni-NTA beads (Thermo Scientific). Beads were then washed with binding buffer and bound proteins were eluted by heating in SDS sample buffer. Western blots were then performed to assess pull-down of TM (monoclonal antibody D-3 from Santa Cruz Biotechnology) and co-precipitation of thrombin (goat polyclonal antibody from R&D Systems).

### Isolation of mouse platelets for western blot

All mouse experiments were approved by Regeneron’s Institutional Animal Care and Use Committee. All mouse experiments were performed in accordance with relevant guidelines and regulations.

Blood was collected from C57Bl/6 mice by cardiac puncture and expelled into tubes containing EDTA and immediately mixed. Blood was then centrifuged at 300 × g for 10 minutes. The supernatant (platelet-rich plasma) was collected and spun at 2,000 × g for 10 minutes and the pellet was then resuspended directly in SDS sample buffer and heated. Aliquots of platelet lysate were then used in western blots, probing for PF4 (goat polyclonal from R&D Systems raised against mouse PF4, used at 1:5000 dilution), Ang1 (rabbit polyclonal generated at QCB for Regeneron, used at 1 μg/ml) or Ang2 (goat polyclonal (C-19) from Santa Cruz Biotechnology, used at 1:200 dilution). The Ang1 and Ang2 antibodies recognize both the mouse and human proteins.

### Blood collection and serum/plasma preparation

For serum preparation, blood was collected by cardiac puncture and expelled into serum separator tubes (BD Biosciences). Following a 30 minute incubation to allow for blood clotting, samples were centrifuged at 2,000 × g for 10 minutes and the serum layer was collected and stored at −80 °C.

For plasma preparation, blood was collected by cardiac puncture, expelled into EDTA tubes (BD Biosciences), mixed and then centrifuged at 2,000 × g at 4 °C for 10 minutes. The supernatant (platelet-poor plasma) was collected and centrifuged again at 2,000 × g for 10 minutes. The plasma supernatant was collected and either used immediately or stored in aliquots at −80 °C.

For tail nick experiments to assess the levels of Ang1 in wound blood, C57Bl/6 mice were placed under a heating lamp for approximately 5 minutes. Mice were then placed in a restrainer and the distal 2 to 4 mm of tail was severed with a single diagonal cut. Blood was collected into heparinized capillary tubes for an initial period of 30 seconds and discarded. Blood was then collected into a second heparinized capillary tube until the tube was full (this took 2–5 minutes). A disposable Pasteur pipette was used to expel the blood into a microfuge tube containing EDTA. Plasma was then prepared as described above. For this experiment, the samples used to assess systemic plasma Ang1 levels were collected by cardiac puncture of naïve animals. To mimic the procedure for preparation of plasma from wound blood, the blood was stored in heparinized capillary tubes for 4–5 minutes before being expelled into a microfuge tube containing EDTA.

### Thrombin Treatment

C57Bl/6 mice were injected via the tail vein with 10 units of human thrombin (Sigma) in a volume of 100 μl. At 10 minutes after injection, blood was drawn by cardiac puncture and plasma was prepared for use in Ang1 and PF4 ELISAs. Any bloods that were difficult to draw were discarded and plasma samples in which the Ang1 level was >30 ng/ml were not used in the analysis, as these samples appeared to be outliers and likely had some degree of platelet activation and Ang1 release *ex vivo*.

### ELISAs

#### Ang1 ELISA

96-well ELISA plates (Corning) were coated overnight at 4 °C with 10 μg/ml of His-tagged Tie2 extracellular domain. Wells were washed 3 times with PBS + 0.05% Tween 20 (PBST) and blocked for 2 hours at RT with PBS + 3% BSA. Wells were washed again and incubated for 1 hour with 100 μl of protein standard (recombinant human Ang1 from R&D Systems) or plasma/serum samples diluted in PBST + 3% BSA. Plasma samples were diluted 1:5, serum samples were diluted 1:20. Wells were then washed and incubated at RT for 1 hour with 100 μl of biotinylated goat anti-Ang1 antibody (R&D Systems, catalog number BAF923) at 0.2 μg/ml. Wells were then washed and incubated in 100 μl of straptavidin-HRP (R&D Systems) at 1:200 dilution for 20 minutes at RT. Wells were then washed and incubated for 20 minutes at RT with 100 μl of substrate solution (R&D Systems, catalog number DY999) protected from light. 50 μl of 1 M sulfuric acid was added to stop the reactions and OD values were read at 450 nm with subtraction at 540 nm.

PF4 ELISA was performed according to the protocol outlined in the mouse CXCL4/PF4 DuoSet (R&D Systems, catalog number DY595). Plasma samples were diluted 1:10 or 1:50 and serum samples were diluted 1:2000.

### Measurement of bleeding times

*Angpt1* whole-body inducible knockout mice were generated using a reverse-tetracycline-controlled transactivator (rtTA) system driven by the ubiquitous ROSA26 promoter as previously described^70^. *Angpt1* was deleted at mid gestation by addition of doxycycline hyclate (0.5%, Sigma Aldrich) to the drinking water of pregnant dams from embryonic day 16.5 until postnatal day 14. *Angpt1* ^flox/flox^ littermate mice lacking the ROSA26^rtTA^ transgene, and thus protected from doxycycline-mediated gene excision, were used as controls. Mice were genotyped by PCR as previously described^70^.

To measure bleeding time, a 3 mm segment from the tail tip was removed with a razor blade and the tail was immediately immersed in 37° PBS. Animals with excessive movement (1 control and 2 mutants) were excluded from the study as the wound was reopened from contact with the side of the beaker.

### Data availability statement

No datasets were generated during the current study. All data generated during this study are included in this published article (and its Supplementary Information files).

## Electronic supplementary material

Supplementary Information
