# Supplementary material for: Angiopoietins bind thrombomodulin and inhibit its function as a thrombin cofactor
Source: Sci Rep. 2018 Jan 11;8:505. doi: 10.1038/s41598-017-18912-8 (PMC5765006; doi:10.1038/s41598-017-18912-8)

Supplementary Information

Angiopoietins bind thrombomodulin and inhibit its function as a thrombin cofactor

Christopher Daly[1]*, Xiaozhong Qian[1]*, Carla Castanaro[1], Elizabeth Pasnikowski[1], Xiabo Jiang[1], Benjamin R. Thomson[2,3], Susan E. Quaggin[2,3], Nicholas Papadopoulos[1], Yang Wei[1], John S. Rudge[1], Gavin Thurston[1], George D. Yancopoulos[1] and Samuel Davis[1]*

* These authors contributed equally to the manuscript

1) Regeneron Pharmaceuticals, Inc., 777 Old Saw Mill River Road, Tarrytown, New York 10591

2) Feinberg Cardiovascular Research Institute, Northwestern University Feinberg School of Medicine, Chicago, Illinois, USA.

3) Division of Nephrology/Hypertension, Northwestern University Feinberg School of Medicine, Chicago, Illinois, USA.

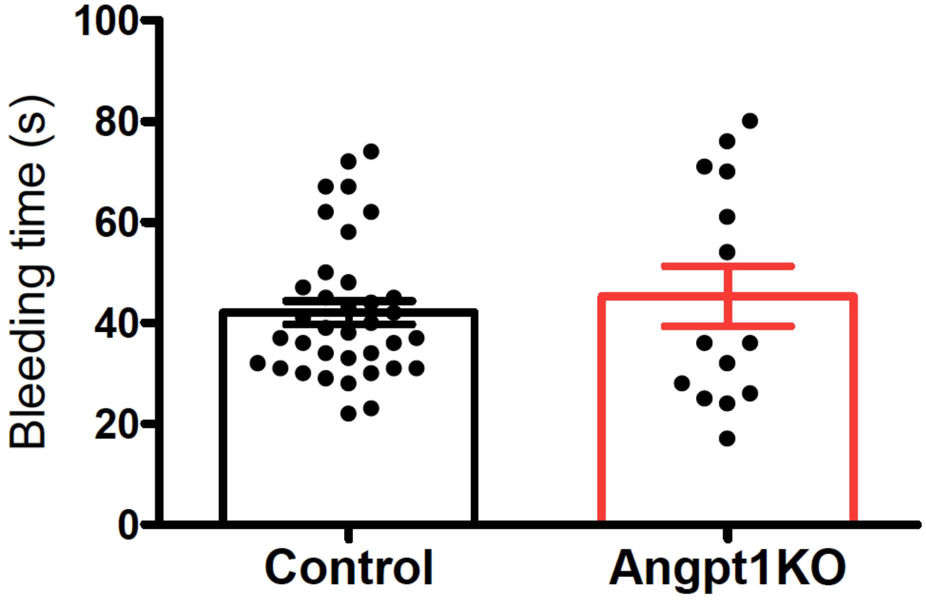

Supplementary Figure 1: Conditional *Angpt1* knockout mice and littermate controls do not exhibit a significant difference in bleeding time.

Tail tip bleeding times were measured (see Methods for details) in a cohort of 6 month-old whole-body *Angpt1* knockout mice (n = 16) and littermate controls (n = 37).

western blot images showing larger gel areas

Fig 1b – TM

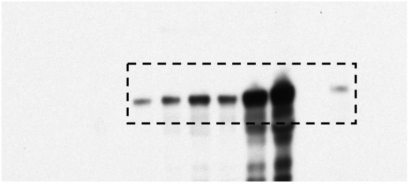

Fig 1b – Tie2

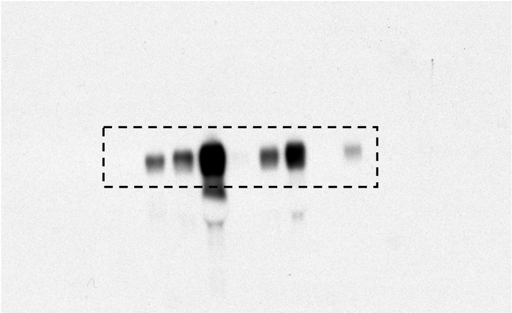

Fig 1c – TM western
(Ang2 binding)

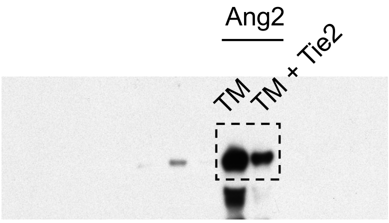

Fig 1c – TM western
(Ang1 binding)

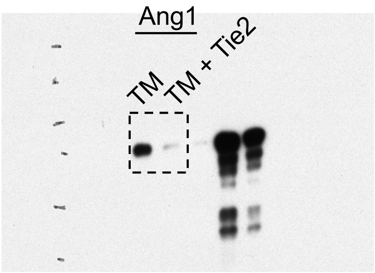

Fig 1c – Tie2 western
(Ang1 binding)

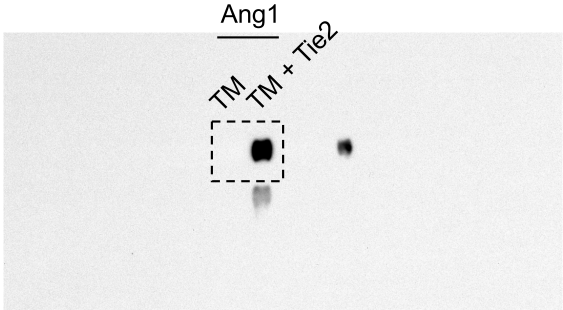

Fig 1c – Tie2 western
(Ang2 binding)

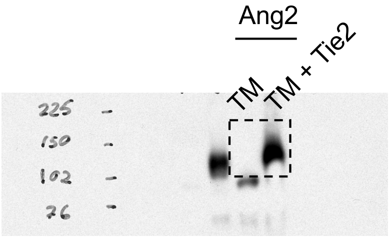

western blot images showing larger gel areas

Fig 4a thrombin

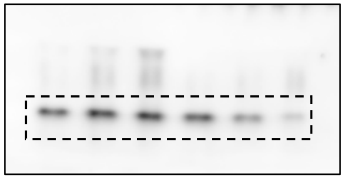

Fig 4a TM

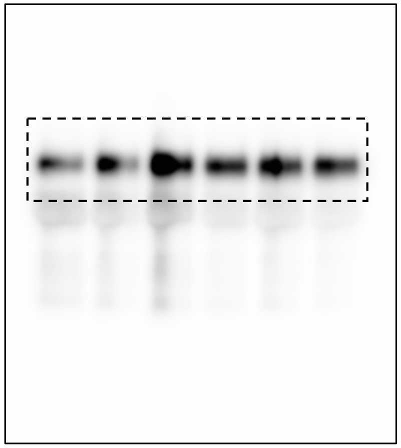

Fig 4b thrombin

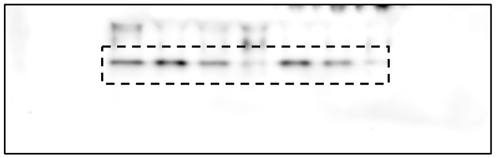

Fig 4b TM

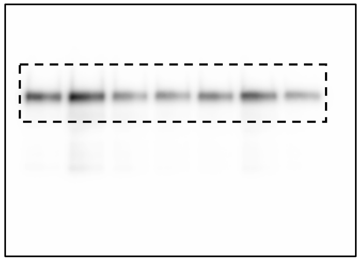

Fig 5a PF4

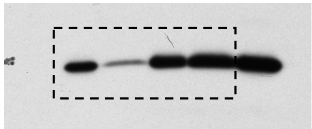

Fig 5a Ang1

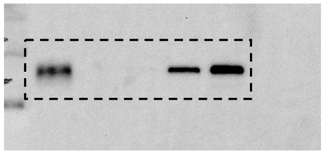

Fig 5a Ang2

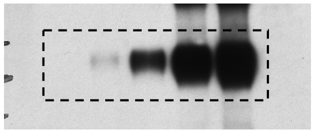

Supplement: Supplementary file 1 — Supplementary Information [file 41598_2017_18912_MOESM1_ESM.pdf]
